# AKT Inhibitors Promote Cell Death in Cervical Cancer through Disruption of mTOR Signaling and Glucose Uptake

**DOI:** 10.1371/journal.pone.0092948

**Published:** 2014-04-04

**Authors:** Ramachandran Rashmi, Carl DeSelm, Cynthia Helms, Anne Bowcock, Buck E. Rogers, Janet Rader, Perry W. Grigsby, Julie K. Schwarz

**Affiliations:** 1 Department of Radiation Oncology, Washington University School of Medicine, St. Louis, Missouri, United States of America; 2 Department of Genetics, Washington University School of Medicine, St. Louis, Missouri, United States of America; 3 Department of Cell Biology and Physiology, Washington University School of Medicine, St. Louis, Missouri, United States of America; 4 Division of Nuclear Medicine, Mallinckrodt Institute of Radiology, Washington University School of Medicine, St. Louis, Missouri, United States of America; 5 Division of Gynecologic Oncology, Washington University School of Medicine, St. Louis, Missouri, United States of America; 6 Alvin J. Siteman Cancer Center, Washington University School of Medicine, St. Louis, Missouri, United States of America; 7 Dept of Obstetrics and Gynecology, Medical College of Wisconsin, Milwaukee, Wisconsin, United States of America; H.Lee Moffitt Cancer Center & Research Institute, United States of America

## Abstract

**Background:**

PI3K/AKT pathway alterations are associated with incomplete response to chemoradiation in human cervical cancer. This study was performed to test for mutations in the PI3K pathway and to evaluate the effects of AKT inhibitors on glucose uptake and cell viability.

**Experimental Design:**

Mutational analysis of DNA from 140 pretreatment tumor biopsies and 8 human cervical cancer cell lines was performed. C33A cells (*PIK3CAR88Q* and PTENR233*) were treated with increasing concentrations of two allosteric AKT inhibitors (SC-66 and MK-2206) with or without the glucose analogue 2-deoxyglucose (2-DG). Cell viability and activation status of the AKT/mTOR pathway were determined in response to the treatment. Glucose uptake was evaluated by incubation with ^18^F-fluorodeoxyglucose (FDG). Cell migration was assessed by scratch assay.

**Results:**

Activating *PIK3CA* (E545K, E542K) and inactivating *PTEN* (R233*) mutations were identified in human cervical cancer. SC-66 effectively inhibited AKT, mTOR and mTOR substrates in C33A cells. SC-66 inhibited glucose uptake via reduced delivery of Glut1 and Glut4 to the cell membrane. SC-66 (1 µg/ml-56%) and MK-2206 (30 µM-49%) treatment decreased cell viability through a non-apoptotic mechanism. Decreases in cell viability were enhanced when AKT inhibitors were combined with 2-DG. The scratch assay showed a substantial reduction in cell migration upon SC-66 treatment.

**Conclusions:**

The mutational spectrum of the PI3K/AKT pathway in cervical cancer is complex. AKT inhibitors effectively block mTORC1/2, decrease glucose uptake, glycolysis, and decrease cell viability *in vitro*. These results suggest that AKT inhibitors may improve response to chemoradiation in cervical cancer.

## Introduction

Globally, cervical cancer is the third most common female cancer, and it ranks fourth in terms of mortality [1]. Concurrent chemoradiation (pelvic irradiation with the concurrent administration of cisplatin chemotherapy) is the standard of care for patients with locally advanced cervical cancer. We have previously demonstrated that the results of post-therapy [^18^F]-fluoro-deoxy-glucose-positron emission tomography (FDG-PET) are predictive of progression-free and overall survival outcomes after chemoradiation [2]–[4]. Presently there are no effective treatment options available for patients whose tumors fail to respond to traditional chemoradiation.

Recently, we identified PI3K/AKT pathway alterations in tumors from patients with a positive post-therapy FDG-PET. We also observed high p-AKT expression in pre-treatment biopsy samples, and patients whose tumors expressed high levels of p-AKT had decreased survival outcomes and increased metastatic disease after standard chemoradiation [5]. Genetic alterations leading to activation of the PI3K/AKT/mTOR pathway are associated with treatment resistance in variety of solid tumors [6]. Several PI3K/AKT inhibitors have been evaluated in clinical trials for breast and other cancers with positive responses in patients with PI3K/AKT alterations [7]. There are reports suggesting that cancers with *PIK3CA* mutations are more sensitive to AKT or PI3K/mTOR inhibitors [8], [9].

We hypothesized that PI3K/AKT inhibitors will improve response to chemoradiation in cervical tumors with PI3K/AKT pathway alterations. To test for mutations in the PI3K/AKT pathway, we analyzed 140 pretreatment cervical tumor biopsies and 8 human cervical cancer cell lines [10]. We then selected the cervical cancer cell line C33A, which is mutated for both *PIK3CA* and *PTEN* (*PIK3CA* R88Q, *PTEN* R233*) and expresses high levels of p-AKT at baseline, to assess the response to two allosteric AKT inhibitors, SC-66 and MK-2206.

## Materials and Methods

### Patients

The study population included 140 patients prospectively enrolled into tumor banking studies at the time of diagnosis of cervical cancer (March 1998 through July 2011). Approval from the institutional Human Research Protection Office was obtained for this study, and all patients signed informed consent. Clinical follow-up including FDG-PET imaging was performed for each patient according to institutional guidelines as previously described [3]. At the time of last follow up, 76 patients had no evidence of disease, and 8 patients were alive with disease; 7 patients had died due to intercurrent illness; 2 patients had died due to treatment-related toxicity, and 47 patients had died due to cervical cancer. Median follow up for patients alive at the time of last follow up was 41 months (range 4 to 161 months).

### Statistical analysis

Survival and tumor recurrence were measured from the completion of treatment. The Kaplan-Meier (product-limit) method was used to derive estimates of survival [11]. Tests of the equivalence of estimates of survival between patient groups were performed by the generalized Wilcoxon log-rank test. Statview version 5.0.1 software (SAS Institute Inc., Cary, NC) was used for the analysis.

### Mutational analysis using MALDI-TOF

Tumor biopsies were sectioned and reviewed for tumor cell content as previously described [5]. Tumor DNA was prepared using standard methods by the Washington University Tissue Procurement Core Facility. Assays for a subset of 32 selected oncogenic mutations (*AKT1*, *AKT2*, *PIK3CA* and *PTEN*) from [10] were redesigned into three genotyping multiplexes, using Sequenom's Assay Designer software, version 3.1.2.2. (Sequenom Inc, San Diego, CA). The multiplexes were designed to use the iPLEX chemistry. The Sequenom MassARRAY system (http://www.sequenom.com) employs MALDI-TOF (Matrix-Assisted Laser Desorption/Ionization – Time of Flight) mass spectrometry to measure mass differences following single-base additions to extension primers. Spectral peaks corresponding to expected masses for each extended primer are transformed into sample genotype calls. Using the standard iPLEX protocol, the Genotyping core at Washington University (St. Louis, MO, USA) processed 15 ng sample DNA per multiplex through the MassARRAY system. Peak areas representing normal and mutant base additions were obtained from each sample's mass spectrum. A mutation was considered present in a sample when the mutant peak was responsible for 25% or more of the combined peak areas, and absent when the mutant peak area was less than 25% of the total. List of OncoMap mutations that were tested in our multiplexes are OM_00970-AKT1-E17K, OM_00032-AKT2-S302G,OM_00033-AKT2-R371H, OM_00241-PIK3CA-R88Q,OM_00242-PIK3CA-N345K,OM_00243-PIK3CA-C420R, OM_00246A-PIK3CA-E545K,OM_00248-PIK3CA-H701P, OM_00249-PIK3CA-H1047L, OM_00250A-PIK3CA-H1047R, OM_00250B-PIK3CA-H1047R,OM_00251-PIK3CA-H1047Y,OM_01017-PIK3CA-E545A, OM_01018B-PIK3CA-N1068fs*4,OM_0102-PIK3CA-Y1021C,OM_00839-PTEN-R173C,OM_00840-PTEN-R173H,OM_00841-PTEN-R233*,OM_00842-PTEN-R335*, OM_01038-PTEN-K267fs*9 OM_01039-PTEN-V317fs*3, OM_01069-PTEN-K6fs*4.

### Cell culture and Reagents

Cervical cancer cell lines were maintained in IMDM media (Life Technologies, CA) with 10% heat inactivated FBS and incubated at 37°C in 5% CO_2_. SC-66 was purchased from Biovision (Milpitas, CA) and MK-2206 from Selleck Chemicals (Houston, TX). 2-Deoxy glucose, protease and phosphatase inhibitor cocktails were purchased from Sigma (Saint Louis, MO). All drugs for cell culture were dissolved in dimethyl sulfoxide (DMSO, Sigma). siRNA oligos against *AKT1*, *AKT2* and *RICTOR* were purchased from Sigma (Saint Louis, MO).

### Western blotting and membrane isolation

Phosphorylation of AKT and downstream targets of AKT and mTOR pathway with or without SC-66 (6–10 µg/ml) and MK-2206 (0–2.5 µM) were determined by western blotting with primary antibodies against phosphorylated and total forms of mTOR, p70s6k, 4E-BP1, S6, GSK3-β, FOXO pAKT^Thr308^, pAKT^Thr450^ and pAKT^Ser473^ (1∶1000; Cell Signaling Technology, MA), total forms of AKT, mTOR and 4-EBP1 (1∶1000, Cell Signaling Technology, MA), total forms of p70s6k and β-Actin HRP from Santa Cruz Biotechnology, CA and total forms of PRAS40 and FOXO from millipore (1∶5000, Santa Cruz Biotechnology,CA). β-Actin was used as the internal control. Blots were probed with HRP-conjugated anti-rabbit (Cell Signaling Technology, Beverly, MA) or anti-mouse polyclonal IgG secondary antibodies (Santa Cruz Biotechnology, CA) for 1 h at RT. For detection Pierce West Dura substrate (Pierce Biotechnology) was used according to manufacturer's protocol and exposed on X-ray film.

### Cell viability and Annexin staining

For the cell viability assay C33A cells were treated with the allosteric AKT inhibitors SC-66 (0.0001 µg/ml–5 µg/ml) and MK-2206 (125 nM-30 µM) with or without the glucose analogue 2-deoxyglucose (2-DG) (5–20 mM) using dose titration and time courses. For siRNA experiments, C33A cells were transiently transfected and assessed for protein expression after 48 hours. Cell viability was tested using Alamar Blue from Life Technologies, according to manufacturer's instructions. Annexin/7-AAD staining was performed 24 h post-treatment, using a kit from BD, Biosciences following manufacturer's instructions, and cells were analyzed by flow cytometry.

### FDG uptake assays

The FDG uptake assay was performed as described previously [5]. Briefly, cells were seeded and pretreated with the block (Cytochalasin B) for 30 min followed by AKT inhibitors for an additional 30 min. After this, ^18^FDG was added to glucose free medium for 1 h. Cells were washed, harvested and counted on a gamma counter.

### Immunofluorescence

In a chamber slide (8-well) 25,000 cells were seeded and treated with SC-66 1 µg/ml for 3 h and fixed using 4% p-formaldehyde from Electron Microscopy Sciences (Hatfield, PA) for 10 minutes. The slides were then blocked in 5% normal goat serum (Jackson ImmumoResearch, West Grove, PA) for 1 h, followed by extensive PBS washes. Then primary antibodies Glut1 and Glut4 (Abcam, Cambridge, MA) were added followed by secondary antibody conjugate with Alexa Fluor 488 (Life Technologies, Inc., Grand Island, NY). The slides were finally mounted using Prolong Gold anti-fade (Life technologies, Inc. Grand Island, NY).

### Metabolic assays

Lactate assay was performed using a kit from (Sigma, Saint Louis, MO) according to manufacturer's instructions using culture media collected after deproteinization using 10 kDa spin column filters. ATP and NADP/NADPH assays were performed using the commercially available fluorometric kits from (Abcam, Cambridge, MA) according to the manufacturer's instructions using cell lysates.

### Wound healing assay

One million C33A cells were plated in a 35 mm tissue culture dish and grown to confluence. Two parallel scratches were made with a 200 µL pipette tip per dish and the scratch width was measured to be the baseline [12]. The wound width was measured at a minimum of six different points for each wound. SC-66 (1 and 2.5 µg/ml) and MK-2206 (2.5 and 5 µM) were added for 24 hr. The width of the scratches was measured using Qcapture Pro software and viewed using OLYMPUS 1×70 microscope. Percent wound healing was calculated by dividing the scratch width after drug addition by the control width minus 100%. The results presented are mean ± SEM.

## Results

### PI3K/AKT/mTOR pathway is active in cervical cancer cell lines

**Figure 1:**
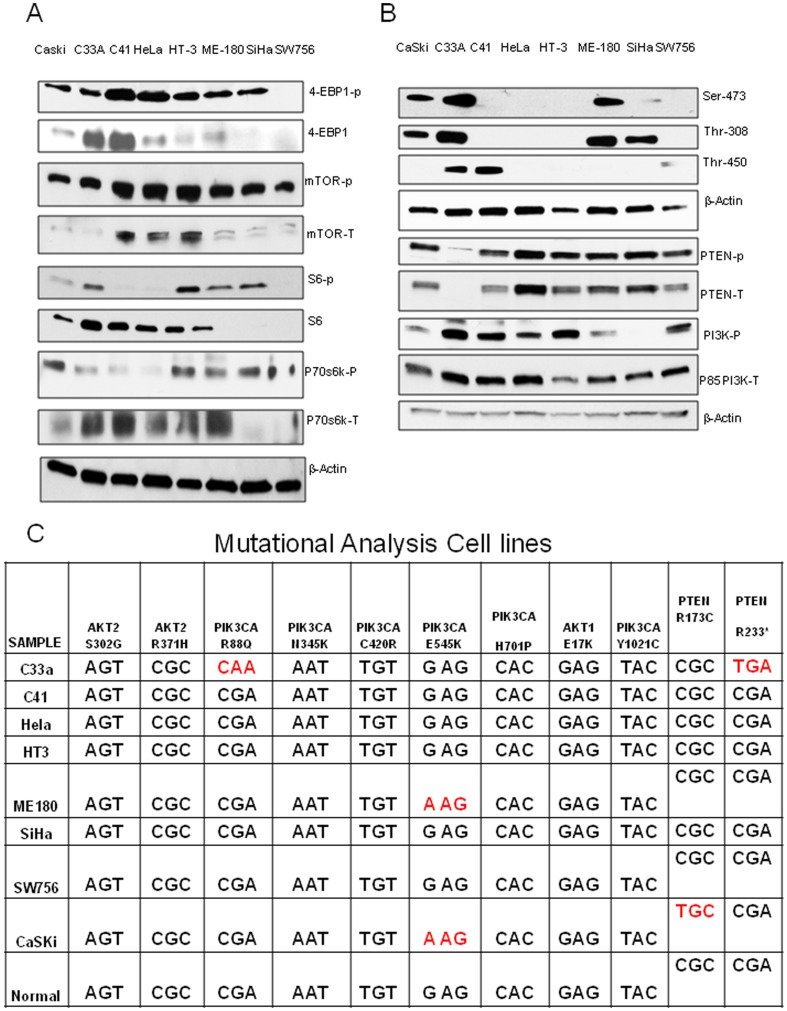
PI3K/AKT pathway analysis in cervical cancer cell lines. (A–B) mTOR pathway components and phosphorylated forms of AKT were tested using commercially available antibodies on eight cervical cancer cell lysates prepared without any treatment. (C) *PIK3CA*, *AKT*, and *PTEN* gene mutational status of 8 human cervical cancer cell lines.

A panel of human cervical cancer cell lines was tested for the expression pattern and activation status of PI3K/AKT/mTOR pathway molecules. Cell lysates were prepared without any treatment and baseline western blots were performed. P70S6K, the marker for activation of the mTOR pathway, was phosphorylated in majority of the cell lines except for HeLa, C41 and C33A where it was found to be weakly phosphorylated (Fig. 1A). Phosphorylation of 4E-BP1 and S6 were found to be low in the majority of the cell lines studied. In SiHa and SW756, the expression levels of non-phosphorylated forms of mTOR, p70s6k, 4E-BP1 and S6 were low compared to the other cell lines. On the other hand, phosphorylation of mTOR was found to be similar across the cell lines (Fig. 1A). Baseline expression of phosphorylated forms of AKT such as Ser473, Thr308 and Thr450 were determined. C33A expressed all the three forms of p-AKT. To determine the status of upstream regulators of AKT such as PI3K and PTEN, baseline p-PI3K and p-PTEN levels were examined. Phosphorylated PTEN level was similar across the cell lines, except for C33A where the level of total PTEN band was minimal. PI3K was activated in the majority of cell lines studied here. SiHa exhibited very low PI3K activation (Fig. 1B). All these results suggest that cervical cancer cell lines have activated mTOR pathway under basal conditions and wide variations existed in p-AKT levels.

### Sequenom mutational analysis of PIK3CA, PTEN and AKT genes in cervical cancer

To test for mutations in the PI3K/AKT pathway, we carried out a Sequenom mutational analysis for oncogenic mutations in genes *PIK3CA*, *PTEN* and *AKT*. We did not detect any *AKT1*or *AKT2* mutations in the cervical cancer cell lines. C33A harbored an R88Q *PIK3CA* mutation. R88Q is an activating mutation found in the ABD domain of the p110α subunit of the *PIK3CA* gene. This defect is associated with enhanced enzymatic activation of PI3K protein and AKT activity *in vitro* [13], [14]. We found that the E545K *PIK3CA* mutation was present in ME-180 and CaSki cells (Fig. 1C). The *PIK3CA* E545K mutation is an activating mutation in the helical domain of p110α subunit of PI3K protein. This mutation is known to confer enhanced kinase activity and to constitutively activate AKT [15]. We also found an R173C *PTEN* gene mutation in CaSki and a *PTEN* R233* mutation in C33A cells (Fig. 1C). The *PTEN* R173C mutation is associated with decreased phosphatase activity against PIP_3_ [16]. The *PTEN* R233* mutation in exon 7 induces a premature stop codon into the gene, which explains the absence of *PTEN* protein expression in C33A cells [17] (Fig. 1B).

**Figure 2:**
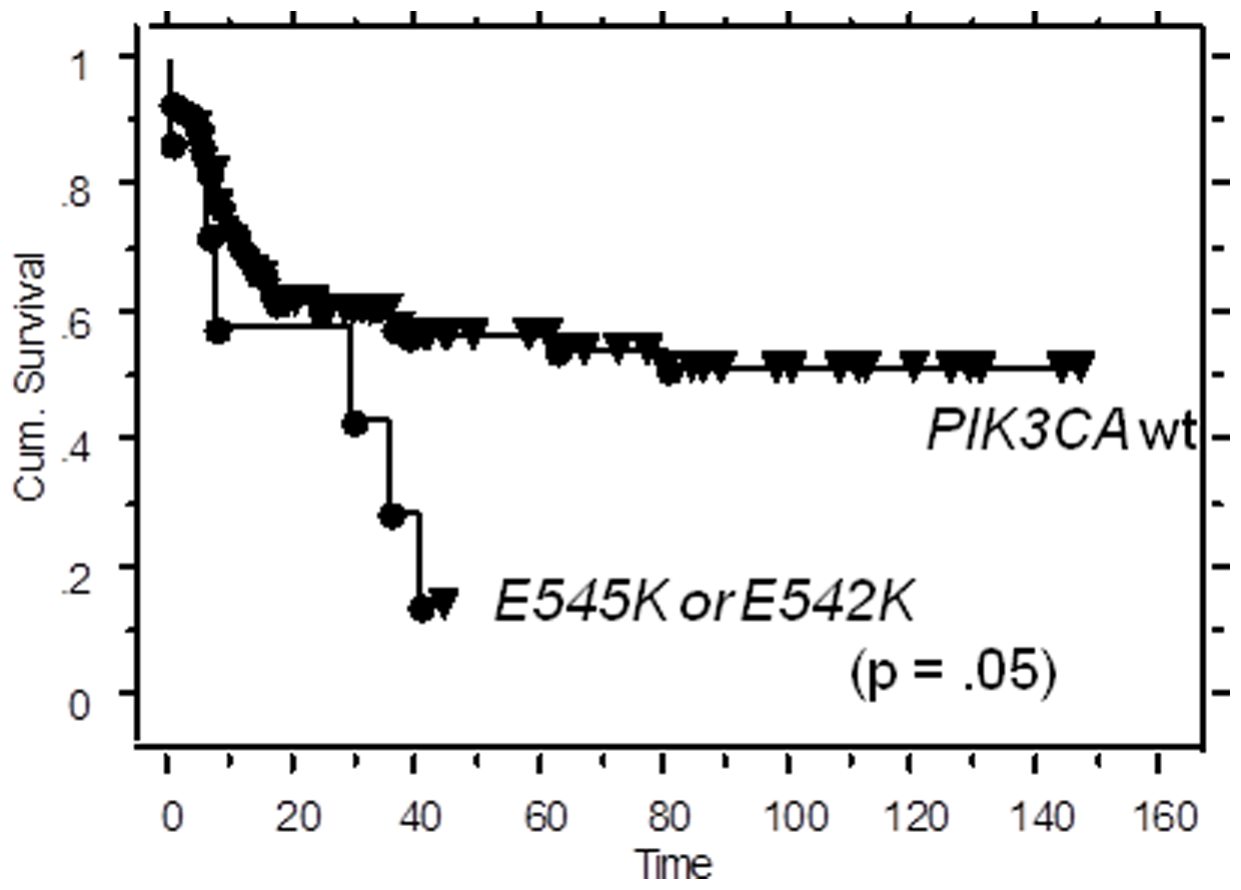
Cervical cancer patients with E545K or E542K mutant tumors have inferior survival outcomes after standard chemoradiation (cisplatin plus pelvic RT). Kaplan Meier curve for progression-free survival for cervical cancer patients with wild type PIK3CA versus E545K or E542K mutant tumors (p = .05).

Using the Sequenom assay, we then tested for mutations in *PIK3CA*, *AKT* and *PTEN* genes in 140 pretreatment biopsies collected at our tumor bank. We did not detect any assayed mutation in the *AKT* gene in our patient population. We found that tumors in 7 out of 140 patients harbored a *PIK3CA* E545K mutation and 1 out of 140 had a *PIK3CA* E542K mutation. We also found that 1 tumor harbored a *PTEN* R233* mutation. Patients with E545K and E542K mutations in *PIK3CA* were found to display poor prognosis and shorter disease free survival after standard chemoradiation (pelvic irradiation and concurrent cisplatin chemotherapy) (p = 0.05, Fig. 2).

### AKT inhibitors SC-66 and MK-2206 induce non-apoptotic cell death in PIK3CA and PTEN mutant C33A cells

**Figure 3:**
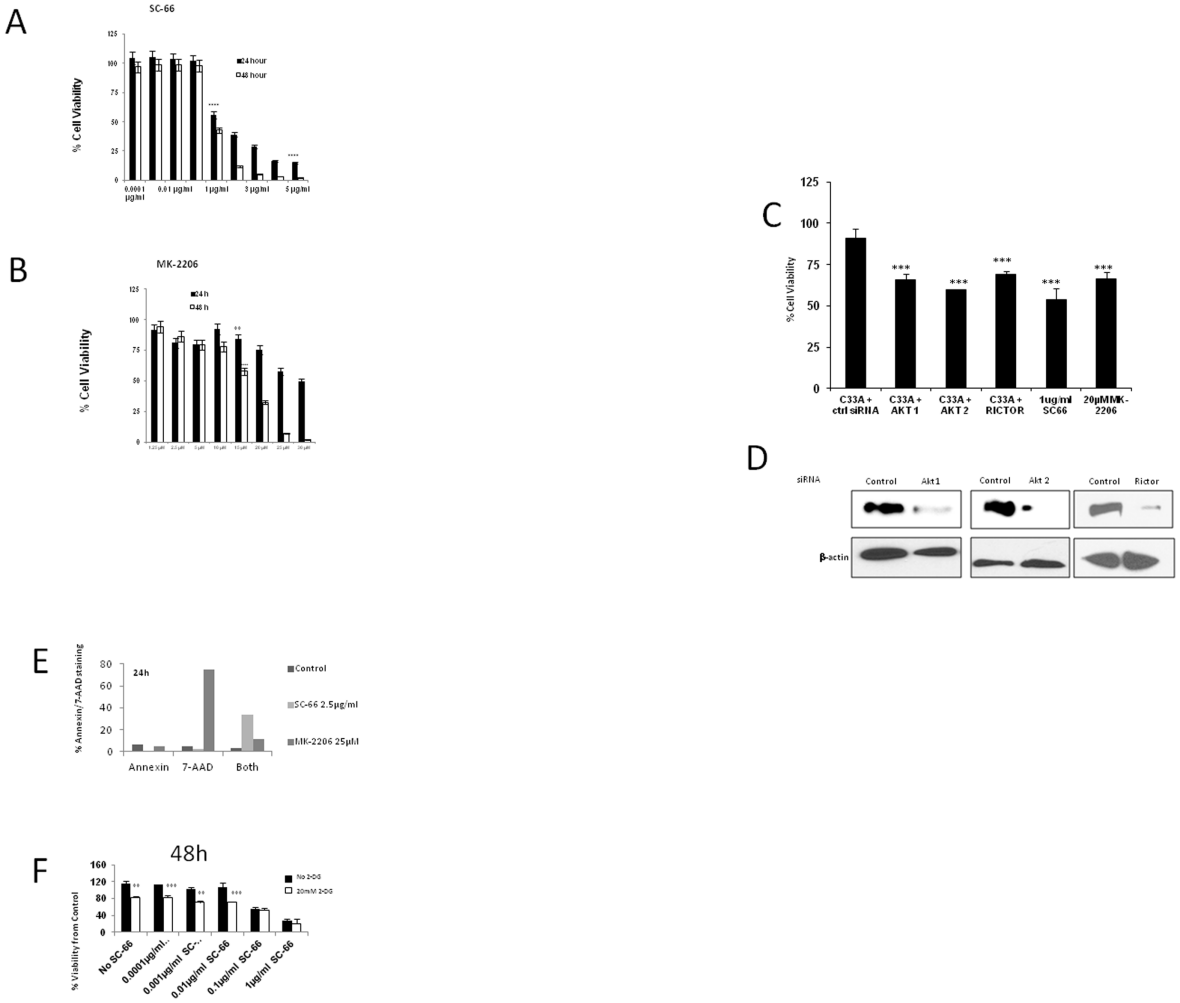
Effects of AKT inhibitors on cell viability. (A–B) C33A cells were seeded on to 48 well plates and treated with increasing doses of SC-66 (0.0001–5 µg/ml) and MK-2206 (1.25–30 µM) in triplicates for 24 and 48 hrs. Viability was measured using Alamar Blue. Percent viability was calculated based on vehicle treated controls. (C) C33A cells were seeded on to 48 well plates and transfected with oligos against *AKT1*, *AKT2* and *RICTOR* and treated with SC-66 (1 µg/ml) and MK-2206 (20 µM) in triplicates for 24. Viability was measured using Alamar Blue. Percent viability was calculated based on vehicle treated controls and control siRNA transfected controls, p<0.001 for the comparison of control siRNA versus siRNA for *AKT1*, *AKT2* and *RICTOR*, SC-661 µg/ml, MK-2206 20 µM. (D) C33A cells were seeded on to 48 well plates and transfected with oligos against *AKT1*, *AKT2* and *RICTOR* and lysates were prepared after 48 h and western blots were performed. (E) C33A cells were treated with SC-66 (2.5 µg/ml) and MK-2206 25 µM for 24 h then stained with Annexin/7-AAD and analyzed by flow cytometry. The graph represents % cell viability. (F) C33A cells were treated with SC-66 (0.0001 µg/ml–0.1 µg/ml) with or without 20 mM 2-DG for 48 h.

Using C33A cells as a model for PI3K/AKT mutant cervical cancer, we determined whether tumor cell survival was dependent on AKT signaling. C33A cells were incubated with increasing doses of SC-66 and MK-2206 and the viability was determined after 24 and 48 hrs. Cell viability decreased starting from 1 µg/ml of SC-66 after 24 and 48 hrs, to 55% and 43% respectively. The viability at 5 µg/ml of SC-66 was found to be 15% after 24 h (Fig. 3A). C33A cells were also sensitive to another allosteric AKT inhibitor, MK-2206. Cell viability was found to decrease starting with the concentration of 15 µM (58%) and decreasing to 2% by 48 h (Fig. 3B). To confirm that affects on cell viability were due to AKT inhibition rather than off target effects of SC-66 and MK-2206, siRNA experiments were performed. As shown in Figure 3C, C33A cell viability decreased to a similar extent when cells were transfected with siRNAs resulting in knockdown of *AKT1*, *AKT2*, and *RICTOR* (Fig. 3D).

To explore the mechanism through which AKT inhibitors induce cell death, we performed Annexin/7-AAD staining. Upon SC-66 (2.5 µg/ml) and MK-2206 (25 µM) treatment there were very few cells with Annexin only staining and the fraction of cells with both staining was 35% and less than 20%, respectively. 7-AAD only staining was close to 80% in MK-2206 treated cells (Fig. 3E). To further link effects of SC-66 through glucose uptake inhibition, we combined SC-66 with 2-deoxy glucose (2-DG), a competitive inhibitor of glucose uptake. By 48 hr after treatment with SC-66 (0.01 µg/ml) the viability was at 107%, but with the addition of 2-DG, cell viability decreased to 72% p<0.001 (Fig. 3F). MK-2206 exhibited synergistic effects with 2-DG. After 24 hr, the viability of MK-2206 treated cells was 78% which decreased up to 40% after addition of 20 mM 2-DG (p<0.001, Data A in File S1).

### SC-66 and MK-2206 inhibited mTOR/AKT pathway effectively in C33A cells

**Figure 4:**
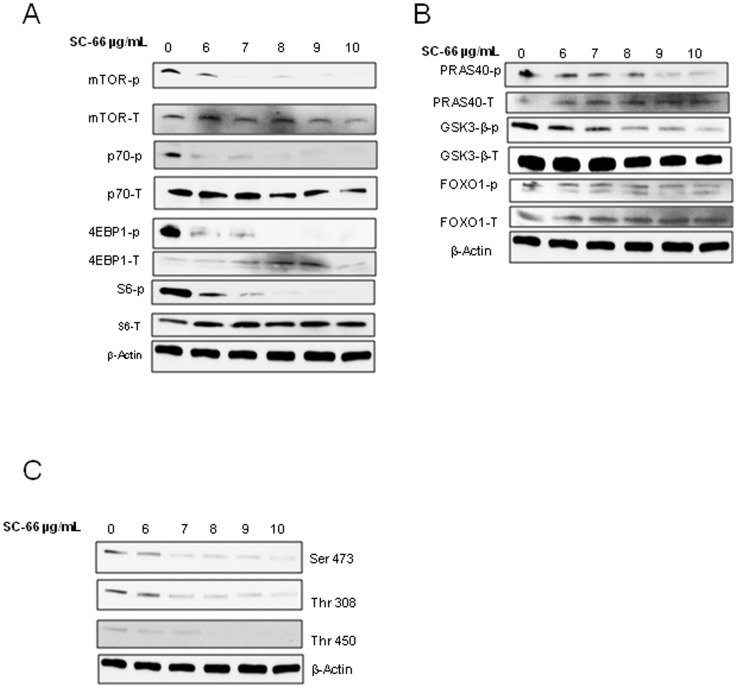
Effect of SC-66 on mTOR signaling. (A–C) C33A cells were treated with increasing concentrations of SC-66(6–10 µg/ml) for 3 h and lysates were prepared for western blot.

To explore the effects of AKT inhibition on mTOR and its downstream targets in C33A cells, we examined the phosphorylation status of mTOR pathway components by Western blot and used p70S6K as a marker for mTOR activation [18]. SC-66 completely inhibited p70S6K phosphorylation by 3 hours (Fig. 4A). MK-2206 inhibited p70S6K activation but there was slight reactivation by 4 h. MK-2206 inhibited mTOR pathway components such as mTOR, 4E-BP1 and S6 effectively by 2 hours. MK-2206 inhibition of mTOR pathway appears to be transient as p70s6k was still active after 4 h (Data D in File S3). SC-66 and MK-2206 effectively inhibited all the three phosphorylated forms of AKT (Thr308, Thr450 and Ser 473) in a dose dependant manner suggesting that MK-2206 acts primarily through mTORC1 (Fig. 4C and Data F in File S3). This is supported by the observation that SC-66 was more effective in inhibiting AKT substrates such as PRAS 40, GSK3-β and FOXO1(Fig. 4B) compared to MK-2206, particularly PRAS40 which is in mTORC1 complex [19] (Data E in File S3). P70S6K was phosphorylated after 4 h of MK-2206 treatment suggesting the mTOR pathway inhibition was transient (Supplemental data).

Rapamycin, an mTOR inhibitor, relieves feedback inhibitions and induces AKT Ser473 phosphorylation in an mTORC2-dependent manner leading to further AKT activation [20]. To test for this effect using our inhibitors we performed a longer incubation of the cells with SC-66 for 18–24 h. We found that p70S6K, the marker of mTOR activation, was decreased even after 18 and 24 hrs treatment. SC-66 treatment decreased activation of AKT substrates, Thr308 and Thr450 by18 and 24 hours. Thr308 levels did go up compared to 3 h sample but still displayed a decreasing trend at 18–24 h (data not shown). All these results suggest that SC-66 effectively inhibited both mTORC1/2 and AKT. MK-2206 was found to be acting mainly through mTORC1 pathway with slight reactivation of the pathway after 4 hours.

### SC-66 inhibited glucose uptake and membrane translocation of glucose transporters

**Figure 5:**
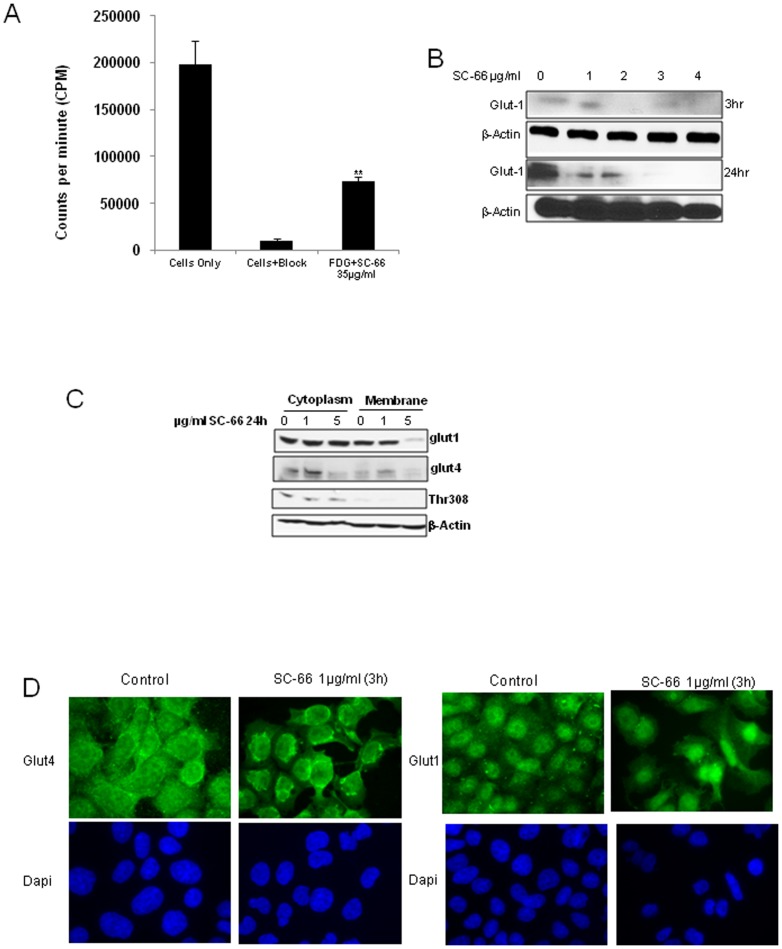
Effects of SC-66 on glucose transport. A) C33A cells were treated with SC-66 (35 µg/ml) or block (cytochalasin B) for 30 minutes prior to incubation with ^18^F-fluorodeoxyglucose as described in the methods section. The graph represents counts per minute values, p<0.01 for the comparison of FDG alone (cells only) versus FDG + SC-66. B) C33A cells were treated with SC-66 (0–5 µg/ml) for 3 and 24 h and Glut1 levels were analyzed by western blot. C) C33A cells were treated with SC-66 (0, 1 and 5 µg/ml) for 24 h. Membrane and cytosol fractions were prepared using a kit (MemPER) from Peirce Biotechnology. These subcellular fractions were then mixed with sample buffer and incubated at 65°C for 20 mins before loading onto the gels for western blot for Glut1 and Glut4. D) Immunofluorescence was performed on C33A cells after treating them with SC-66 (1 µg/ml) for 3 hours in a chamber slide.

Glucose uptake was tested by performing *in vitro* FDG uptake assays in the presence and absence of the SC-66 (35 µg/ml). We found that SC-66 inhibited glucose uptake significantly as evidenced by reduced counts per minute (Fig. 5A). Further we determined the effect of SC-66 on Glut1 and Glut4 translocation to the membrane. For this a membrane cytoplasm fractionation was carried out after treating cells with SC-66 (5 µg/ml) for 24 h. SC-66 inhibited Glut1 and Glut4 translocation from the cytoplasm to the membrane as determined by Western blot (Fig. 5C). We confirmed this with immunofluorescent staining (Fig. 5D). All these results suggest that mTOR inhibition by SC-66 resulted in decreased glucose uptake through Glut1 and Glut4 retention within cytoplasm.

### AKT inhibitor SC-66 inhibits glycolysis

**Figure 6:**
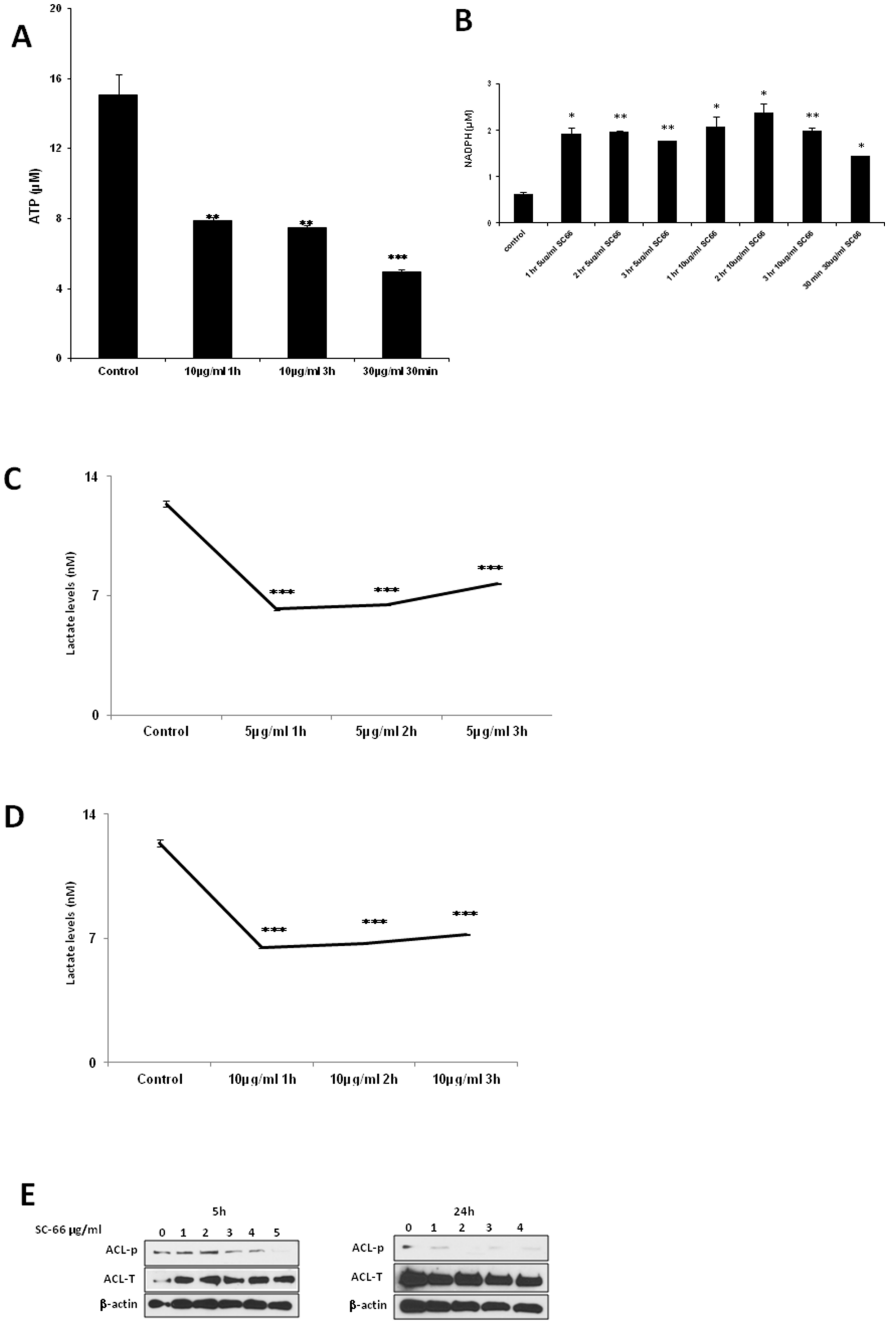
Effects of SC-66 on glucose metabolism. (A–B) C33A cells were seeded in T 25 cm^2^ tissue culture flasks, treated with SC-66 and intracellular NADPH levels and ATP were determined. The graph represents ATP and NADPH µM levels based on standard curve, p<0.01 for the comparison of control versus SC-66 10 µg/ml 1 and 3 h and p<0.001 for control versus 30 µg/ml for ATP levels; p<0.01 for the comparison of control versus SC-66 5 µg/ml 3 h and p<0.01 control versus 10 µg/ml 3 h for NADPH levels. (C–D) C33A cells were seeded in T 25 cm^2^ tissue culture flasks, treated with SC-66 and excreted lactate levels were measured in nM concentrations using a standard curve, p<0.001 for the comparison of control versus SC-66 5 and10 µg/ml. (E) C33A cells were treated with increasing concentrations of SC-66 (0–5 µg/ml) for 5 h and 24 h and lysates were prepared for western blot.

To determine whether AKT inhibition resulted in reduced glycolysis, we measured ATP and NADPH levels after SC-66 treatment. We found that ATP levels were decreased significantly after SC-66 treatment, and NADPH levels were increased, suggesting that alternative pathways of glucose metabolism, such as the pentose phosphate shunt, may be more active in C33A cells when AKT signaling is suppressed (Fig. 6A and 6B). Total lactate levels were also decreased in C33A cells after treatment with SC-66 (Fig. 6C and 6D). All these results indicate that inhibition of AKT suppresses glycolysis in C33A cells. To evaluate for downstream effects on tumor cell metabolism, we monitored the activation status of a substrate of AKT, ATP-citrate lyase (ACL) after SC-66 treatment. C33A cells were incubated with increasing doses of SC-66 and western blots were performed. SC-66 inhibited ACL phosphorylation by 5 and 24 hours, suggesting that inhibition of AKT may also influence other aspects of tumor cell metabolism, including lipid synthesis (Fig. 6E).

### SC-66 reduces migration of C33A cells in vitro

**Figure 7:**
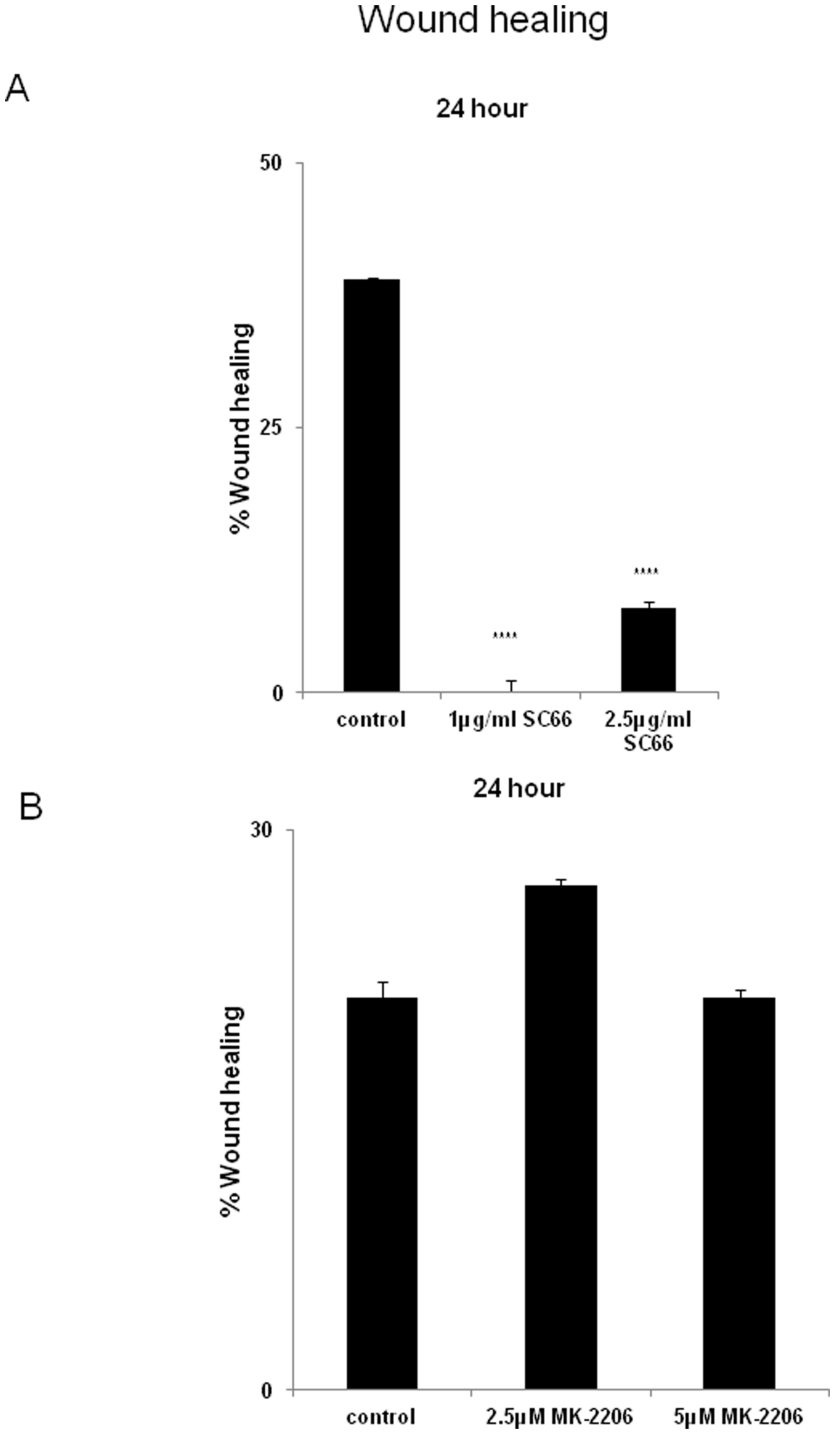
Effects of SC-66 and MK-2206 on cell migration. C33A cells were treated with A) SC-66 (1 and 2.5 µg/ml) and B) MK-2206 (2.5 and 5 µM) for 24 h. Percent wound healing was calculated as described in methods section, p<0.0001 for the comparison of control versus 1 ug SC-66 and p<0.0001 for control versus 2.5 ug SC-66.

There are reports showing the role of AKT in the metastatic process including cell migration and invasion [21]. To study the effects of SC-66 and MK-2206 on cell migration we treated cells with 1 µg/ml SC-66 and 2.5 µM MK-2206 for 24 h and performed a scratch assay. We found that SC-66 inhibited the migration of cells about 50% compared to control whereas MK-2206 did not have any effect (Fig. 7).

## Discussion

In this study, we performed mutational analysis of 140 pretreatment tumor biopsies and 8 human cervical cancer cell lines to screen for mutations in the PI3K/AKT pathway. This is the first study, to our knowledge, to comprehensively analyze mutations in the PI3K/AKT pathway in human cervical cancer. We identified multiple mutations in the PI3K/AKT pathway in human cervical cancer specimens, including activating mutations in *PIK3CA* (E545K, E542K) and inactivating mutations in *PTEN* (R233*). Mutational analysis of cervical cancer cell lines revealed additional defects. C33A cells have both an R233* *PTEN* mutation and an R88Q *PIK3CA* mutation [22].

The present study was also designed to test the hypothesis that cervical cancer cells with altered AKT activation would be sensitive to AKT inhibitors. SC-66 and MK-2206 effectively induced cell death in C33A cells through a non-apoptotic mechanism. SC-66 was found to be a potent mTORC1/2 inhibitor. SC-66 effectively inhibited p70s6k and 4E-BP1 mTORC1 substrates, in addition to inhibiting Ser473 and Thr308 phosphorylation of AKT, and activation of AKT substrates such as PRAS40, GSK3-β and FOXO. SC-66 displayed synergistic effects with 2-DG, and SC-66 inhibited further downstream events such as translocation of glucose transporters to the membrane which resulted in decreased glucose uptake. In addition, inhibition of AKT reduced glycolysis as evidenced by decreased ATP and lactate levels, and increased activity of alternative metabolic pathways resulting in increased cellular NADPH. These results suggest that AKT inhibitors decrease cervical cancer viability by interfering with cellular glucose metabolism. We hypothesize that cervical cancers with PI3K/AKT pathway alterations are dependent upon high rates of glucose uptake and glycolysis for survival. It should be noted that activation of Akt by *PTEN* loss and/or *PIK3CA* mutations would bypass the need for the activation of growth factor receptors (i.e. IGF-1R) to initiate the PI3K/Akt/mTOR signaling cascade. In this manner, cervical cancer cells are able to upregulate glucose import and metabolism even in the absence of the appropriate external signals.

Previously it has been shown that inhibition of mTORC2 leads to rapid inhibition of AKT Ser473 phosphorylation, which accelerates the destabilization process of Thr308 site phosphorylation [23]. In accordance with this, we also observed that SC-66 mediated a concomitant reduction in phosphorylation of Ser473 and Thr308 in C33A cells. Earlier work from others suggested that dephosphorylation of AKT at Thr308 site leads to more profound inhibition of AKT function than would be seen from dephosphorylation of AKT at Ser473 alone [23]. Thr308 is a residue in a key T-loop of the AKT protein and considered as a better indicator of AKT kinase activity. There are discordant data on the Ser473 phosphorylation and AKT kinase activity [24], [25]. Our results show that SC-66 inhibits all of the three phosphorylated forms of AKT.

There are reports that mTORC2 is required for development of certain cancers with *PTEN* loss [26]. C33A cells are *PTEN* defective, and we found that SC-66 is a potent mTORC2 inhibitor. The *PTEN* R233* mutation found in C33A cells can lead to greater intracellular accumulation of PIP_3_ resulting in enhanced PI3K signaling and PDK-1-mediated AKT Thr308 phosphorylation [25], [27], [28]. We speculate that SC-66 exerts its inhibitory effect on AKT Thr308 phosphorylation indirectly by acting as a PDK-1 inhibitor. SC-66 might not be as efficient as an inhibitor of PDK-1 as it is for mTOR and AKT, and this explains the slightly higher Thr308 levels observed after longer incubation. Moreover, we observed that SC-66 is a potent cell death inducer by 24 h, thus reactivation of AKT may not be an issue *in vivo*. In concordance with this notion, we found that in mice treated with a combination of cisplatin and SC-66, all p-AKT forms were inhibited compared to the monotherapy counterparts. This is true for the mTOR pathway components such as p70s6k, 4E-BP1 and S6 as well (data not shown).

AKT2 stimulates glucose uptake through the glucose transporter 4 (Glut4) translocation to membrane via a substrate called Syntaxin interacting protein (Synip) [29]. Membrane localization of AKT2 is a pre-requisite for Glut4 translocation in response to glucose uptake [30]. SC-66 effectively inhibited Glut1 and Glut4 membrane translocation, a key step mediated by AKT for glucose uptake. AKT activation leads to increased Glut1 expression and translocation to the membrane resulting in greater glucose uptake [31]. Our results indicate that SC-66 also inhibited Glut1 protein expression, suggesting that in C33A cells Glut1 expression is AKT-dependent.

In our study we also show that 2-DG enhanced MK-2206- and SC-66-induced cell death. It is known that glucose deprivation mimicked by glycolytic inhibitors causes cytotoxicity by inducing oxidative stress in human cancer cells [32], and cisplatin is known to disrupt thiol metabolism and to enhance oxidative stress [33]. We hypothesize that combining cisplatin, SC-66/MK-2206 and 2-DG will display synergistic effects (Data B in File S1 and Data C in File S2). This synergy could be explained as disruption of cellular thiol pools and enhancement of oxidative stress by cisplatin and 2-DG respectively. Interestingly, treatment with Akt inhibitors does not render C33A cells resistant to death associated with glucose withdrawal, and we have observed induction of autophagy in response to MK2206 treatment. Additional studies will be needed to further characterize the role of autophagy in C33A cell survival.

Our preliminary studies based on Sequenom assays confirm that *PIK3CA* and *PTEN* genes are mutated in cervical cancer patients with poor progression free survival after standard chemoradiation. Frequency of the PI3K/AKT pathway mutations in human tumors widely vary in different types of cancer [10]. Recently, McIntyre *et al* described that *PIK3CA* E545K mutational status was associated with response to chemoradiation in cervical cancer patients [34]. A more comprehensive analysis of mutations in a larger cohort of patients is required to establish the link between PI3K/AKT pathway mutations and treatment outcome. If these results are validated, PI3K/AKT pathway mutations may be used in the future to select tumors at risk for treatment failure using standard chemoradiation (pelvic irradiation and concurrent administration of cisplatin chemotherapy).Our results suggest that AKT inhibitors could improve response to chemoradiation in cervical cancer for appropriately selected patients. It is possible that the mutations reported here (PIK3CAE545K, PIK3CAE542K and PTEN R233*) may used in the future to select patients for targeted treatment with PI3K/AKT pathway inhibitors. Experiments are ongoing to determine the appropriate timing of AKT inhibition in the context of pelvic irradiation on chemotherapy.

## Supporting Information

File S1 Contains Data A and B.
(TIF)

File S2 Contains Data C.
(TIF)

File S3 Contains Data D, E, and F.
(TIF)
