# Correction: Correction: AKT Inhibitors Promote Cell Death in Cervical Cancer through Disruption of mTOR Signaling and Glucose Uptake

**DOI:** 10.1371/journal.pone.0155168

**Published:** 2016-05-05

**Authors:** The PLOS ONE Staff

The sixth author’s name is spelled incorrectly. The correct name is: Janet S. Rader.
